# Cockroaches, locusts, and envenomating arthropods: a promising source of antimicrobials

**DOI:** 10.22038/IJBMS.2018.30442.7339

**Published:** 2018-09

**Authors:** Mahnoor Ummul-Warah Faateemah Zehra Mosaheb, Naveed Ahmed Khan, Ruqaiyyah Siddiqui

**Affiliations:** 1 Department of Biological Sciences, School of Science and Technology, Sunway University, Selangor, Malaysia

**Keywords:** Antibacterial agents, Anti-infective agents Antimicrobial peptides Communicable diseases Drug resistance, Invertebrate peptides

## Abstract

**Objective(s):**

To present a brief overview of various natural sources of antimicrobials with the aim of highlighting invertebrates living in polluted environments as additional sources of antimicrobials.

**Materials and Methods:**

A PubMed search using antibacterials, antimicrobials, invertebrates, and natural products as keywords was carried out. In addition, we consulted conference proceedings, original unpublished research undertaken in our laboratories, and discussions in specific forums.

**Results:**

Representative of a stupefying 95% of the fauna, invertebrates are fascinating organisms which have evolved strategies to survive germ-infested environments, yet they have largely been ignored. Since invertebrates such as cockroaches inhabit hazardous environments which are rampant with pathogens, they must have developed defense mechanisms to circumvent infections. This is corroborated by the presence of antimicrobial molecules in the nervous systems and hemolymph of cockroaches. Antimicrobial compounds have also been unraveled from the nervous, adipose, and salivary glandular tissues of locusts. Interestingly, the venoms of arthropods including ants, scorpions, and spiders harbor toxins, but also possess multiple antimicrobials.

**Conclusion:**

These findings have rekindled the hopes for newer and enhanced therapeutic agents derived from a plentiful and diverse resource to combat fatal infectious diseases. Such antimicrobials from unusual sources can potentially be translated into clinical practice, however intensive research is needed over the next several years to realize these expectations.

## Introduction

Ranked as the second leading killer, infectious diseases pose a serious threat to humankind, accounting for an estimated 17 million deaths (1). These statistics comprise a daily toll of 50,000 men, women, and children dying, despite advances in antimicrobial chemotherapy. Contrary to the wide belief that infectious diseases have been largely alleviated, malaria, cholera, and tuberculosis alone remain significant threats, while HIV/AIDS, Ebola, dengue, and Zika pose a major risk to human health (2, 3). For instance, in 2015, 10.4 million cases with 1.4 million deaths have been associated with tuberculosis alone (4). In part, this is attributed to the emergence of multi-drug resistant *Mycobacterium tuberculosis*, displaying resistance against anti-tuberculosis drugs such as isoniazid and rifampicin (5). Antimicrobial resistance has engendered a crisis amidst the soaring infection rates with stunted antimicrobial discovery (6). The increase in multi-drug resistance of pathogens, hampered the progress of antibiotic discovery, by adding to the existing financial and temporal burden in devising new antimicrobial chemotherapy. The currently available antibiotics encompass mainly structural analogs of antibiotics from the 1940-1980s era, often with sub-optimal efficacy (7). Hence, there is an urgent need to discover novel and effective antibiotics. This has sparked the antibiotic hunt from natural sources. Plants and marine algae have often been acclaimed for their beneficial antimicrobial properties (8). However, instead of focusing on an extinguishable antimicrobial source from the flora, it would be interesting to consider the fauna as well. Since invertebrates represent a staggering 95% of the fauna (9) and have existed for millions of years in hazardous environments, they are promising candidates. These creatures are believed to have developed antimicrobials to protect themselves from the pathogenic microbes (10). 

 Some of the invertebrates thrive under hazardous conditions known to humans. For instance, cockroaches dwell in polluted environments, infested with multi-resistant pathogens. This led to the hypothesis that invertebrates possess antimicrobials as part of their defense mechanisms (11). De facto, the innate immunity of invertebrates is highly responsive to bacterial challenges, producing humoral antimicrobials as part of its defense system (12). Furthermore, venoms of invertebrates including scorpions, spiders, and ants possess antimicrobial activity (13, 14). Thus, it is reasonable to explore invertebrates to recruit valuable antimicrobial products of their defense mechanisms.

## Methods

A PubMed search using “antibacterials” and “antimicrobials’ combined with “invertebrates” and “natural products” as keywords was carried out. In addition, we consulted conference proceedings, original unpublished research undertaken in our laboratories, and discussions in forums (e.g., American Society for Microbiology Annual Meeting in 2017, European Society of Clinical Microbiology and Infectious Diseases in 2017, the Free-Living Amoebae Meetings in 2017, and Spanish Parasitology Society in 2017).

## Results

***Locusts and cockroaches***

*Periplaneta americana* cockroach (Linnaeus) is an ancient species of flying cockroaches which coexist with humans in urbanized milieus (15). Cockroaches occupy pathogen-infested environments and thrive, suggesting the presence of antibacterial molecules (11). In support, cockroach brain lysates exhibited potent bactericidal effects against methicillin-resistant *Staphylococcus aureus* and pathogenic *Escherichia coli* K1 (11). Size-exclusion analysis depicted low molecular-weight compounds below 10 kDa were present in the brain extracts of *P. americana.* Furthermore, the brain lysates depicted selective toxicity against pathogens but non-toxic to human cells, as analyzed through cytotoxicity assays on human brain microvascular endothelial cells (7). Preliminary studies unraveled novel neuropeptides in the organ lysates (11). Smaller antimicrobial peptides arise from cleavage of these neuropeptides, notably from proenkephalin and prodermaseptin (12). These comprise 24-34 subunit amphiphilic helical cysteine-free, 20-46 amino acid cysteine-rich, or 10-13 residue lipophilic peptides. (16). The LC-MS identified many biologically-active compounds with useful therapeutic effect (7). The compounds identified from brain and hemolymph extracts revealed antimicrobial activity against a multitude of Gram-positive and Gram-negative bacteria, several viruses and fungi, and *Candida albicans* (7). Moreover, isoquinoline and thiazine groups, chromene-derived compounds, pyrrole-containing entities, sulfonamides, furanones, and flavanones, which protect against inflammation, oxidative stress, cancer, and several bacterial infections were identified (7). Seraj and colleagues endeavored to isolate the antimicrobial peptides in the hemolymph of *P. americana* cockroach when immune-challenged by *E. coli* (17). Through the disk-diffusion technique, the researchers reported antibacterial activity in the hemolymph of *P. americana* against *Bacillus* species, *S. aureus, *and both drug-resistant and susceptible *E. coli* strains (18). The purified active fractions from hemolymph yielded 2 bands of approximate molecular mass of 61 and 67 kDa, which bear antibacterial effects at 2.87 µg, compared to common antibiotics which mount similar antibacterial effects at 10 µg (17). 

The migrating insects, *Locusta migratoria* represent ubiquitous pests that rampage fields (19). They are a widespread nuisance in agriculture, implying that the outsourcing of these notorious pests, towards antimicrobial discovery, might resolve dual issues. Since the nervous system governs the vital functions of organisms, it could potentially represent the site of antimicrobials (11). The antibacterial activity of organ lysates from *L. migratoria*, including the nervous, hemolymph, adipose, and muscular tissues was determined (20). The effects were tested on *S. epidermidis, S. aureus,* and drug-resistant pathogens including methicillin-resistant *S. aureus* (MRSA) and pathogenic *E. coli* K1 (20). Bacteria incubated with PBS alone showed approx. 10^6^ c.f.u. while bacteria incubated with brain lysates showed zero colonies (20). In contrast, other tissue extracts such as fat bodies and muscles showed no effects and bacterial CFU were recovered in numbers similar to bacteria incubated with the solvent alone. The dose-dependent antibacterial assays reflected that the brain lysates of *L. migratoria* retain their antibacterial properties at concentrations of less than 5 µg (12). Furthermore, the high bactericidal property was retained even when the brain lysates were heated, but not when exposed to denaturing agents like SDS. Additionally, these antimicrobial peptides bear selective toxicity to both Gram-positive and Gram-negative bacteria, without affecting eukaryotic cells (20). In another study, immune-challenged *L. migratoria* were surveyed for antimicrobial activity in the fat body lysates or salivary glands and palps (19). The locusts were harnessed at the gregarious phase during their metamorphosis and injected with *Serratia marcescens*, *Nosema locustae,* and *Metarhizium anisopliae* to stimulate the innate immunity and assess the locust’s potential in antimicrobial production. Antibacterial assays against *S. aureus* and *E. coli* revealed the antimicrobial activity of the lysates in the immune-challenged locusts. Reverse-transcription of the isolated RNA indicated the up-regulated expression of 3 novel defensins in immune-challenged locusts including LmDEF-5, LmDEF-4, and both LmDEF-1 and LmDEF-3 (19). Separation through PAGE yielded bands of 5 and 11 kDa, representative of the small defensin peptides. Lv and colleagues successfully determined the presence of three novel peptides as cysteine-rich defensins and another conserved defensin-like molecule (19). Thus, *L. migratoria* locusts harbor highly-evolved innate immunity to stimulate the production of antimicrobial peptides (19). 

***Venoms of arthropods as antimicrobial source***

Arthropods are ancient creatures that have evolved multiple defense mechanisms against predation, food-chain competition, and pathogenic infection (21). For instance, they resort to the use of stingers and production of venoms, which contain a wealth of amines, alkaloids, enzymes, hydrocarbons, and antimicrobial peptides (22, 23). These findings suggest that the antimicrobial potential of envenomating arthropods is worth investigating. The diverse array of peptides from venoms include 30–50 amino-acid disulfide-containing peptides, specifically acting as ligands to receptors of membrane-transporters to monitor downstream activity (21). Besides, smaller unsulfurylated venom peptides, different primary structures have been categorized as either helical positively-charged or coiled anionic acidic molecules (24). The amphiphilic and positively-charged peptides exhibit antimicrobial activity against bacteria, fungi, as well as viruses (25).

 Recently, four novel antimicrobial peptides, occurring in the venom of *Heterometrus spinifer* scorpion, have been identified. Given their unique sequence, compared to known antimicrobial peptides of the database, they have been coined as HsAp, HsAp 2, HsAp 3, and HsAp4 (14). RNA extracts from venoms of electrocuted scorpions were reverse-transcribed to create a genomic library. Genomic sequencing highlighted that these novel antimicrobial genes are void of non-coding regions, suggesting they are involved in stimulating initial antibacterial responses (14). The cloned antimicrobial molecules were then expressed to analyze the protein separation profile through gel electrophoresis. Bioinformatics studies revealed that the venom antimicrobials from *H. spinifer* have 29 subunits in length, with the 7th to 24th amino acids coiled into an alpha-turn attributed to the amphipathic nature of the peptides (14). 

In as study, the folding of the peptides synthesized based on the natural *H. spinifer* peptides suggested their antimicrobial potential (14). They investigated the antimicrobial activity of the newly-discovered peptides through antibacterial assays against Gram-positive and Gram-negative bacteria, as well as, antifungal assays. The antibacterial property of the peptides was shown through MIC values ranging between 11.8 and 51.2 µM. In fact, the lowest inhibitory value being 11.8 µM for *Bacillus magaterium *highlights the potential of these venom peptides as antimicrobials (14). *Candida tropicalis* was inhibited at a concentration of 48.6 µM, confirming the antifungal potency of novel HsAp peptides (14). 

***Spiders and ants***

Spiders are ranked as a pharmacologically important envenomating arthropod that have widened their territories by harnessing the synthesis of silk and toxin-rich venom (26). It is believed that over 10 million biologically important peptides occur in the spider venom (26). This is attested by the presence of antimicrobial peptides notably, Lycosin II (27). It has depicted no sequence homology to previously identified peptides from other species nor to Lycosin I from the same species (28). Isolation and characterization analysis revealed Lycosin II as a linear primary structure of 21 monomers that forms a positively-charged alpha-helix (27). It bears high antimicrobial potency against antibiotic-resistant bacteria responsible for hospital-acquired infections. In antibacterial assays against 18 strains of *A. baumanii, S. aureus,* and Gram-negative bacteria, Lycosin-II displayed MICs ranging from 3.1 to 25 µM against all the bacteria tested. Additionally, the exposure to Lycosin-II showed bacterial reductions within 10 min (27). A suggested mode of action is interference with the membrane structure resulting in blockage, as analyzed through competition to the membrane-targeting divalent cation, Mg2+ (27). Moreover, the cytotoxicity assay of Lycosin-II demonstrated 20% lysis of erythrocytes at 50 µM, which is twice the recorded MIC value (27). The comparable high MIC and low hemolytic values suggest Lycosin-II in *Lycosa singoriensis *spider venom is more toxic to pathogens than mammalian cells (27). With almost 13,161 species, ants form part of a rich biodiversity of arthropods (29). *Tetramorium bicarinatum* is one of the most widespread envenomating ants from the Formicidae family. The venom of the *T. bicarinatum* ant is thought to contain antimicrobials (23). Recently, a novel antimicrobial peptide from the ant venom has been characterized and coined as bicarinalin. It is non-disulfide linked with a linear primary structure of several positively-charged portions (29). Bicarinalin bears some homology to the pilosulin peptide, characterized from the venom of Myrmecia ants. Two-dimensional Nuclear Magnetic Resonance (NMR) has further portrayed that it comprises a 10-amino acid sequence, bearing alpha helices within terminal disorganized regions. It exhibited antimicrobial activity against a wide range of bacteria, fungi, and parasites within 0.45 (*Staphylococcus xylosus*) to 97.5 μmol/l (*Geotrichum candidum*) (29, 30). Additionally, it depicts no cytotoxic activity against human lymphocytes at low concentrations within 0.066 to 8.5 μmol/l (29). Bicarinalin has the ability to resist proteolytic activity and persist in the body for up to 15 hr (29). 

## Discussion

Infectious diseases pose a significant threat to human health. The development of new antibiotics has suffered a hiatus, in contrast, antimicrobial resistance is soaring, further narrowing down the treatment options. Thus, there is a dire need to tap into novel antimicrobial sources. Antimicrobial research scans multiple natural resources for novel molecules. Since they are primitive organisms with strong defense systems against infections, invertebrates are potentially rich in antimicrobials, particularly antimicrobial peptides (AMPs).

The presence of antimicrobials in invertebrates without stimulation suggests they are intrinsically rich sources of natural antibiotics. Compared to vertebrates, which possess the specific and non-specific branches of immunity, invertebrates rely heavily on the innate immune system (31). The latter encompasses a broad collection of antimicrobial molecules including peptides. These constitutive invertebrate AMPs, usually shorter than 100 subunits, are often categorized as cysteine-free linear structures bearing helices like cecropins, non-linear disulfide-linked such as defensins or drosomycins and proline or glycine-overrepresented peptides, and even lysozymes or secondary metabolites. The invertebrate AMPs are naturally cationic and amphiphilic and usually have small molecules. Such property suggests a potential membrane-targeting mode of action of AMPs. Positively-charged AMPs preferentially bind to the anionic teichoic acids on Gram-positive bacteria or cardiolipins in bacterial membranes (32). This recognition of prokaryotic structure ensures that there is a limited effect on mammalian membranes. It has been postulated that AMPs can disrupt the phospholipid arrangement through channel formation by perpendicular integration (toroidal and barrel-stave model). Otherwise, AMPs may accumulate along the membrane surface and form parallel complexes that mediate pore formation. The perforated membranes lose their elasticity and enable water entry, eventually leading to cell lysis (32). These proposed models of action of AMPs targeting conserved membrane components, and not receptors, lower the risk of bacteria evolving resistance through drug-target modification, thereby augmenting the therapeutic value of AMPs. The small, positively-charged amphipathic nature of AMPs imply they can be easily absorbed across the phospholipid bilayer (33). There is high potential of using invertebrate AMPs as therapeutics given their antibacterial activity against a wide array of both Gram-positive and Gram-negative pathogens. Some AMPs from both arthropod venoms and insect organs even portray antifungal activity. Furthermore, the innate responses of invertebrates enable rapid response of humoral AMPs’ secretion. Interestingly, AMPs retain their efficacy against drug-resistant pathogens. This is attributed to the diverse range and specificity of each AMP that can also aggregate into complexes to overcome microbial resistance (34).

As per any new drug discovery, discovering novel antimicrobial peptides poses a major challenge in the realm of antimicrobial discovery. A significant limitation is the lack of information about the novel peptides discovered since only a few molecules can be isolated from the purified organ lysates or induced hemolymph (35). Even, genetic analysis of AMP gene orthologies among multiple invertebrate species may fail to detect small precursors or post-translationally modified AMPs. With the development of better separation and sequencing tools, there is huge potential to track the wealth of unidentified AMPs from invertebrates. An example is next-generation sequencing platform for whole genome analysis of invertebrates to scrutinize the AMP-encoding genome and transcriptome (36). Besides, the improvements and revolutionization of chromatographic separation techniques coupled with mass-spectrometry and NMR enable the analysis of a wider picture of the antimicrobial structure and content of invertebrates (7). Natural molecules can serve as a scaffold for bulk-synthesis of antimicrobials (36). Besides, the AMP-encoding gene can be cloned into expression vectors and transformed into competent bacteria for large-scale production in bio-fermenters (19). 

Our findings showed that cockroach brain lysates exhibit potent antibacterial activities, were intriguing, and led to fathom the defense mechanisms of cockroaches (7). Given the unsanitary habitats of cockroaches, they serve as carriers of pathogenic organisms, yet they thrive in these conditions. Other than the nervous systems, the hemolymph may represent a potential antimicrobial source. These findings suggest that invertebrates such as cockroaches and locusts (20) respond to pathogen exposure by synthesizing antimicrobials that are effective at low concentrations. Indeed, this poses as a wild-card in antimicrobial research, as hemocyte-mediated immunity can stimulate antimicrobial production (17, 18), which can be tapped as potential novel antibacterial agents. Similarly, broad-spectrum antimicrobial potential of newly discovered peptides (HsAp, HsAp 2, HsAp 3, and HsAp4) (14) isolated from the venom of scorpion species, *H. spinifer* as well as Lycosin-II isolated from spiders (27) suggested a significant opportunity in our search for novel antimicrobials, especially in the treatment of drug-resistant bacteria. 

Bicarinalin isolated from the venom of the envenomating ant species *T. bicarinatum *exhibited potent antimicrobial activity against a wide range of bacteria, fungi, and parasites (29, 30). It is a 10-amino acid sequence, bearing alpha helices within terminal disorganized regions. The proposed mode of action for bicarinalin is membrane disruption, influencing the permeability, as demonstrated through SYTOX green uptake assay (29, 30). It is speculated that bicarinalin interferes with anionic phospholipids of the membrane, as predicted by the mode of action of positively-charged antimicrobial peptides (30). In addition, it exhibits good pharmacokinetic profile suggesting further that it has potential in antimicrobial chemotherapy.

The use of antimicrobial peptides in drug delivery engenders several concerns given that proteinaceous particles may be susceptible to varying pH and enzymes (32). However, this is not consistent for all invertebrate AMPs, as shown by stable bicarinalin from *T. bicarinatum* ants with good pharmacokinetic profile (29). Additionally, packaging unstable AMPs in agents like liposomes can assist in effective delivery (33). The toxicity of venom peptides can be revoked since it is conferred by a distinct peptide (21). Thus, bioconversion of the toxic entities into useful AMPs, or fractionating the AMP out would provide a natural scaffold to synthesize pharmacophores. Moreover, combinational biology of the identified AMPs along with sub-optimal existing drugs may improve the efficacy of antimicrobial action. For instance, combination of Css54 AMP from *Centruroides suffusus* scorpion venom enhanced the bactericidal activity of rifampicin, an anti-tuberculosis drug, showing promising potential of invertebrate AMPs combination with antibiotics (37). The success of membrane-targeting peptides, recently added to the antibiotic repertoire, like the lipopeptide daptomycin, suggests the potential therapeutic value of invertebrate-derived AMPs (38). This reinforces the opportunity in unraveling antibiotics from invertebrates. So far, insect antimicrobial research lies at the *in vitro* experimental stage. Hence, future research will include furthering the antimicrobial research to isolate additional compounds and characterize them. Besides, the experiment can be scaled up to *in vivo* studies to examine the bio prospects of the antimicrobial peptides for interactions in complex biological pathways.

## Conclusion

Invertebrates represent a rich and diverse source of molecules with important antimicrobial potency. The discovery of antimicrobials from the constitutive or immune-stimulated insects such as cockroaches and locusts and envenomating arthropods such as scorpions, spiders, and ants has carved new avenues in antimicrobial research. Several molecules have been identified that exhibit broad-spectrum antimicrobial activities against bacteria, fungi, and parasites. Future studies are needed to determine *in vivo* efficacy of these molecules to allow their development in clinical applications, especially in the treatment of bacterial infections that are increasingly resistant to presently available drugs, however, this will require intensive research in the next few years. With continued efforts, there is huge opportunity to tap into the therapeutic value of these novel antimicrobial peptides to fight against infectious diseases caused by multi-drug resistant pathogens.
